# Therapy to teratology: chronic paternal antioxidant supplementation alters offspring placental architecture and craniofacial morphogenesis in a mouse model

**DOI:** 10.3389/fcell.2025.1697843

**Published:** 2025-12-19

**Authors:** Destani D. Derrico, Katherine Z. Scaturro, Erin E. Murray, Eliezar Guillen, Nathan S. Truss, Katherine A. Fairly, Samantha L. Higgins, Sanat S. Bhadsavle, Michael C. Golding

**Affiliations:** 1 Department of Veterinary Physiology and Pharmacology, College of Veterinary Medicine and Biomedical Sciences, Texas A&M University, College Station, TX, United States; 2 Interdisciplinary Graduate Program in Genetics and Genomics, Texas A&M University, College Station, TX, United States

**Keywords:** antioxidants, paternal effects, developmental toxicology, placenta, craniofacial dysgenesis, epigenetic inheritance, mitochondria, N-acetylcysteine

## Abstract

Oxidative stress is an important regulator of the mammalian epigenome, with redox imbalances triggering stress-responsive epigenetic modifications linked to various diseases. Accordingly, antioxidant therapies are commonly used to reduce oxidative damage and are widely employed in cases of male infertility. Interestingly, in ostensibly healthy males, recent research suggests that antioxidants may have a negative influence on sperm DNA methylation, indicating a potential epigenetic liability. However, whether male antioxidant treatment can induce paternal effects on offspring growth and development remains untested. Here, we employed micro-CT imaging and geometric morphometrics to determine whether chronic antioxidant supplementation in healthy male mice affects placental growth and craniofacial development in their offspring. Adult C57BL/6J male mice were given a six-week preconception regimen of N-acetyl-L-cysteine and selenium, then paired with treatment-naïve females. Although we observed sex-specific changes in the decidua and junctional zone, we did not detect changes in placental weight and efficiency. In contrast, we observed significant changes in facial shape in both male and female offspring, with female offspring exhibiting significant reductions in eye spacing and head area. These changes occurred without any macro changes in paternal metabolic health, indicating that alterations in developmental programming may occur independent of changes in overall health. Our findings highlight the need for caution in the indiscriminate use of antioxidants, showing that supplementation in healthy males is not harmless and that perturbing the paternal redox balance may alter developmental programming and induce teratogenic outcomes.

## 1 Introduction

Oxidative stress is a potent regulator of the epigenetic landscape, influencing DNA methylation, histone modifications, and noncoding RNAs through both direct and indirect mechanisms (Chen and Shen, 2025). These stress-induced epigenetic alterations, in turn, profoundly affect cellular function and have been implicated in the development of a wide range of human diseases, including age-related pathologies, neurodegenerative disorders, cancer, cardiovascular disease, infertility, and developmental disorders, including fetal alcohol spectrum disorders (FASDs) (Terracina et al., 2024). However, a critical yet emerging area of investigation is the extent to which systemic oxidative stress is sensed by the cells of the reproductive tract and whether mitochondrially driven epigenetic changes can transfer through the mammalian germline to influence offspring health.

Research in mammals demonstrates that maternal dietary and environmental stressors promote the intergenerational transmission of adverse changes in mitochondrial function, impacting offspring growth and long-term health. (Mitchell et al., 2009; Saben et al., 2016; Ferey et al., 2019; Marei et al., 2020; Di Berardino et al., 2022). Similarly, work from our laboratory examining chronic paternal alcohol exposure demonstrates the paternal inheritance of adverse impacts on fetal development that strongly resemble the developmental defects observed in FASDs (Golding, 2023). These impairments first appear during fetal development and are associated with altered placental growth and abnormalities in craniofacial development, including changes in facial shape and symmetry (Thomas et al., 2021; Thomas et al., 2022; Roach et al., 2023a; Bhadsavle et al., 2024; Thomas et al., 2023; Higgins et al., 2024). Notably, these developmental outcomes correlate with adverse effects on offspring mitochondrial function, including impaired complex I activity, shifts in the NADH/NAD^+^ ratio, and disrupted cellular metabolism that persist into adulthood and correlate with accelerated biological aging. Remarkably, paternal alcohol use appears to interact with maternal exposures to accelerate these mitochondrial deficits, promoting a sustained pro-inflammatory state, which increases offspring susceptibility to liver disease and hepatocellular carcinoma (Chang et al., 2019a; Chang et al., 2019b; Basel et al., 2024; Basel et al., 2025).

Our ongoing research indicates that chronic paternal alcohol consumption impairs mitochondrial function within the male reproductive tract, correlating with alterations in sperm noncoding RNAs and long-lasting changes in offspring mitochondrial activity (Bedi et al., 2019; Roach et al., 2023b). Given that reactive oxygen species activate multiple epigenetic and stress-response pathways that directly impact male fertility and epigenetic markers in sperm (Bisht et al., 2017), antioxidant supplementation represents a logical intervention that could modify the epigenetic transmission of alcohol-related paternal effects.

Antioxidant therapies aim to mitigate oxidative stress, a condition characterized by an imbalance between reactive oxygen species and the cellular antioxidant defense (Forman and Zhang, 2021). While preclinical studies highlight the potential of antioxidants to mitigate cellular markers of oxidative damage, clinical outcomes have been inconsistent, with some reports even documenting adverse effects (Forman and Zhang, 2021; Al-Madhagi and Masoud, 2024). Indeed, in a recent study by Hug et al., modeling the effects of oxidative stress on the sperm epigenome, antioxidant therapy successfully corrected redox-induced epigenetic alterations. However, strikingly, non-stressed control animals exposed to the same antioxidants developed epigenetic changes of comparable magnitude to those caused by the original stressor (Hug et al., 2024). These findings join a growing body of research suggesting that antioxidant treatments are not innocuous and, in the absence of oxidative stress, may disrupt normal epigenetic programming in the male germline (Moazamian et al., 2025).

Although healthy men commonly use antioxidants through their widespread incorporation into multivitamins and dietary supplements, in the absence of oxidative stress, this indiscriminate use may induce significant epigenetic alterations in the male germline, raising clinical and public health concerns (Li et al., 2022). However, whether antioxidant-induced epimutations transmit to offspring and affect developmental outcomes remains untested. Therefore, as a preliminary step to investigating the ability of antioxidants to modify alcohol-induced paternal effects, we tested the hypothesis that a chronic antioxidant regimen would cause developmental changes in the offspring of non-stressed, antioxidant-treated fathers compared to unexposed controls.

N-acetyl-L-cysteine (NAC) and selenium (Se) are two widely studied antioxidant compounds used to investigate cellular responses to oxidative stress. NAC primarily functions as a precursor to cysteine, a rate-limiting amino acid required for the synthesis of glutathione (GSH), a major intracellular antioxidant. GSH neutralizes reactive oxygen species by donating electrons and converting to the oxidized glutathione disulfide (GSSG) (Ntamo et al., 2021). Similarly, selenium is a critical component of selenoproteins, including glutathione peroxidases (GPx), which catalyze the reduction of hydrogen peroxide and organic hydroperoxides using GSH as a substrate.

NAC and selenium supplementation have demonstrated beneficial effects on markers of male fertility (Ahmadi et al., 2016; Buhling et al., 2019; de Ligny et al., 2022). Further, given that gestational NAC supplementation can attenuate the programmed susceptibility to obesity and insulin resistance in offspring of mothers maintained on a high-fat diet and correct FASD-related craniofacial phenotypes induced by gestational alcohol exposure (Charron et al., 2020; Parnell et al., 2010), we selected these antioxidants for our studies.

## 2 Materials and methods

### 2.1 Ethics and regulatory compliance

We designed our study in accordance with the ARRIVE guidelines (Percie du Sert et al., 2020) and conducted all experiments in compliance with IACUC regulations and the National Research Council’s Guide for the Care and Use of Laboratory Animals, with prior approval from the Texas A&M University IACUC (protocol number 2023-0186).

### 2.2 Animal studies and antioxidant exposures

We utilized male C57BL/6J strain mice (RRID:IMSR_JAX:000664) obtained from a breeder nucleus at the Texas A&M Institute for Genomic Medicine. We maintained males in the TIGM facility on a reverse 12-h light/dark cycle (lights off at 8:30 AM) and fed them a standard chow diet (Catalog# 2019; Teklad Diets, Madison, WI, United States). Beginning on postnatal day 90, we individually housed each male to monitor the dosing of the antioxidant treatment. To help offset the stress of individual housing and minimize the impact of animal stress (Cait et al., 2022), we added shelter tubes (catalog# K3322; Bio-Serv, Flemington, NJ, United States) and additional nestlets to enhance cage enrichment, as described previously (Thomas et al., 2021; Thomas et al., 2022; Thomas et al., 2023).

We initiated the control and antioxidant treatments by exposing control mice to ultrafiltered water, while we exposed experimental mice to an antioxidant mixture comprised of 4.48 mg/mL N-acetyl-L-cysteine (catalog #A7250, Sigma-Aldrich, St. Louis, MO, United States) and 0.448 ug/mL selenium in the form of sodium selenite (catalog #S5261, Sigma-Aldrich, St. Louis, MO, United States). We selected these dosages from previous publications, anticipating maximum daily dosages of 400 mg/kg/day for NAC (Parnell et al., 2010; Marian et al., 2006; Wright et al., 2015) and 0.04 mg/kg/day for selenium (Hu et al., 2018). We added a zero-calorie flavor enhancer (0.0896% solution, Stevia in the Raw®, Cumberland Packing Corp., Brooklyn, NY, United States) to increase the palatability of the antioxidant treatment. Each week, we recorded the weight of each mouse (g) and the total weekly fluid consumption (g). We then quantified weekly fluid consumption by dividing the grams of fluid consumed by the sire’s body weight (g/g).

**FIGURE 1:**
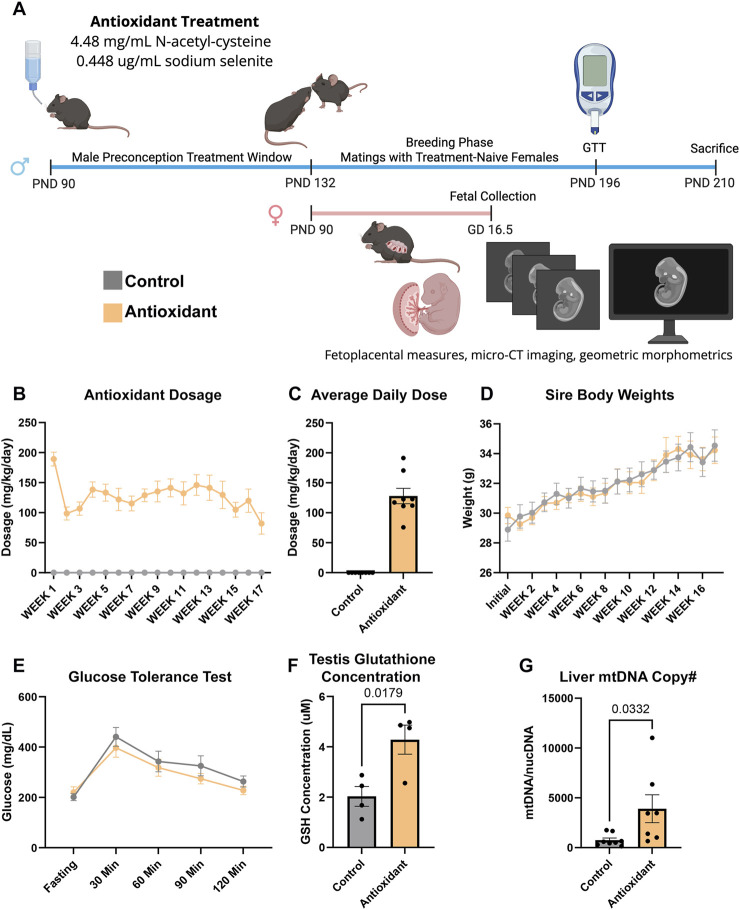
A mouse model to examine the paternal effects of chronic antioxidant supplementation on offspring development. **(A)** Visual representation of the mouse model we used to determine the impacts of chronic paternal antioxidant supplementation on offspring growth and development. **(B)** Average daily NAC dose (n = 8) across the experimental course and **(C)** cumulative average daily dose. **(D)** Comparison of average weekly weight gain between sire treatment groups across the experimental course, including the 6-week preconception and 10-week breeding phases (n = 8). **(E)** Comparison of blood glucose levels during a glucose tolerance test (n = 8). **(F)** Measurement of testicular glutathione concentrations using a colorimetric assay (n = 4). **(G)** qPCR analysis of hepatic mitochondrial DNA copy number between treatments (n = 8). We compared treatment groups using a two-way ANOVA and an unpaired Student’s t-test; data represent mean ± SEM.

We maintained the preconception treatments for 6 weeks, which, in mice, encompasses approximately one complete spermatogenic cycle (Adler, 1996), and continued treatments during the subsequent breeding phase. We paired control and antioxidant-treated males with naïve postnatal day 90 C57BL/6J strain dams, which we obtained from the Texas A&M Institute for Genomic Medicine (Figure 1A). We synchronized female reproductive cycles using the Whitten method (Whitten et al., 1968), then placed one female in the male’s home cage. During this 8-h breeding window, we substituted the antioxidant treatment with filtered water, ensuring females were not exposed to the antioxidant treatment. We confirmed matings by the presence of a vaginal plug, recorded female body weights, and returned females to their original cages. We rested males for 2 weeks, during which they continued the preconception control or antioxidant treatments, and then used them again in a subsequent mating. On gestational day ten, we confirmed pregnancy diagnosis by an increase in body weight of at least 1.8 g. We terminated dams on gestational day 16.5 using carbon dioxide asphyxiation followed by cervical dislocation, dissected the female reproductive tract, and recorded fetoplacental measures. We then collected digital photographs of the front, left, and right profiles of each fetus within each litter. We then either fixed the collected tissue samples in 10% neutral buffered formalin (catalog# 16004-128, VWR, Radnor, PA, United States) or snap-froze the tissues on dry ice and stored them at −80 °C.

### 2.3 Fetal sex determination and molecular analysis

We isolated genomic DNA from the fetal tail using the HotSHOT method (Truett et al., 2000) and determined fetal sex using a PCR-based assay previously described (Thomas et al., 2021). We measured mitochondrial DNA using the PCR assay described previously (Roach et al., 2023b), and cellular glutathione levels using the Glutathione Colorimetric Detection Kit (catalog #K006-H1, Arbor Assays, Ann Arbor, MI, United States).

### 2.4 Placental histological analysis

We examined the impact of preconception paternal antioxidant treatment on placental histology using previously described methods (Thomas et al., 2021; Roach et al., 2023a; Bhadsavle et al., 2024). Briefly, we cut placentae in half and fixed one portion in neutral buffered formalin. We then randomly selected placentae from all litters, stained these samples with phosphotungstic acid to enhance tissue contrast (Lesciotto et al., 2020), and then processed them for Micro-Computed Tomography (micro-CT) imaging. We used Aquasonic Clear Ultrasound Gel (Catalog# 03-08; Parker Labs, Fairfield, NJ, United States) to prevent tissue desiccation during scanning. We imaged the treated samples using a SCANCO vivaCT 40 (SCANCO Medical AG, Brüttisellen, Switzerland) with a 55 kVp voltage x-ray tube and an exposure of 29 μA, yielding an image voxel size of 0.0105 mm^3^ and a resolution of 95.2381 pixels/mm. We then used the open-source medical image analysis software Horos (Version 3.3.6; Nibble Co. LLC, Annapolis, Maryland, United States; https://horosproject.org/) to quantify layer-specific volumes, as described previously (Clercq et al., 2019).

### 2.5 Digital image acquisition and processing

During dissections, we collected digital photographs of the front, left, and right profiles of each fetus within each litter. We then processed the images for morphometric analyses using methods described previously (Thomas et al., 2023; Higgins et al., 2024). Briefly, we imported digital images of the facial profiles into the publicly available software tpsUtil32 ((Rohlf, 2015); version 1.83) to generate TPS files for landmarking. We then used the publicly available software tpsDig2w64 ((Rohlf, 2005) version 2.32) for image analysis by first setting the reference scale bar in the picture to 1 mm and then demarcating the eighteen facial landmarks described previously for the front profile and twenty-two landmarks for the side profiles (Thomas et al., 2023; Higgins et al., 2024) (Supplementary Table S1). To ensure consistency, a single individual (N.S.T.) demarcated the landmarks in each photograph, consistently identifying the exact location and order for each image. To add additional landmarks, we generated the outline around the head using the publicly available program tpsDig2w64 ((Rohlf, 2005) version 2.32), producing a total of 47 landmarks for the front profile and a total of 51 landmarks for the side profiles. In curating this dataset, we named each file with the litter I.D., sex, and uterine position of each fetus. Finally, we used the publicly available program tpsUtil32 ((Rohlf, 2015); version 1.83) to create our final TPS files, inclusive of all landmarks for use in the MorphoJ software for analysis.

### 2.6 Geometric morphometrics and statistical analyses of facial images

We imported the generated TPS files for each fetus into the MorphoJ software ((Klingenberg, 2011) version build 1.07a, Java version 1.8.0_291 (Oracle Corporation)) and conducted geometric morphometric analysis using methods described previously (Thomas et al., 2023; Higgins et al., 2024). Briefly, we added classifiers describing each treatment group and then separately normalized the datasets for scale, rotation, and translation using the Procrustes fit feature (Klingenberg, 2011). We then generated a covariance matrix, which we used to conduct Principal Component Analysis (PCA).

We then used Canonical Variate (CV) analysis to identify differences in facial features between treatments and exported the raw CV scores into the publicly available Paleontological Statistics Software Package for Education and Data Analysis (PAST) analysis software ((Hammer et al., 2025) version 4.03 [https://softfamous.com/postdownload-file/past/18233/13091/.]). We conducted multivariate analyses of the raw CV scores using statistical methods described previously (Zelditch et al., 2012; Attanasio et al., 2013; Wiseman et al., 2021; Geometric Morphometrics Tutorial, 2023). These included the parametric Multivariate analysis of variance (MANOVA), and Nonparametric Analysis of similarities (ANOSIM), and Permutational multivariate analysis of variance (PERMANOVA) tests, followed by Bonferroni correction. We generated the CV lollipop and scatter plots using the graphing features of MorphoJ (Klingenberg, 2011).

### 2.7 Data handling and statistical analysis

We subjected all data generated during this study to the data management practices and statistical analyses described previously (Basel et al., 2024; Basel et al., 2025). Briefly, we recorded our initial observations by hand and then inserted these measurements into Google Sheets or Microsoft Excel. In line with modern statistical reporting (Amrhein et al., 2019), we have moved away from binary significance labels and now interpret p-values as graded evidence against the null hypothesis. We consider p-values below 0.01 to be strong evidence for an effect, while p-values between 0.1 and 0.01 provide moderate evidence of an effect (Muff et al., 2022). Here, we report the exact p-values for each test.

We transferred the collected datasets into GraphPad Prism 10 (RRID:SCR_002798, GraphPad Software Inc., La Jolla, CA, United States). We first employed the ROUT test (Q = 1%) to identify outliers and verified equal variance using either the Brown-Forsythe or F testing. If data passed normality and variance testing (alpha = 0.05), we employed either an unpaired, parametric (two-tailed) t-test or a One-way or Two-way ANOVA. We then used Šídák’s multiple comparisons test or Tukey’s *post hoc* test to compare each treatment to the control. If, however, the collected datasets failed the test for normality or we observed unequal variance, we ran a Kruskal–Wallis test followed by Dunn’s multiple comparisons test. We present detailed descriptions of each statistical test and the sample sizes for each figure in Supplementary Table S2.

## 3 Results

### 3.1 A mouse model to examine the impacts of chronic antioxidant supplementation on offspring fetoplacental growth and craniofacial development

Over the six-week preconception and subsequent ten-week breeding phase (Figure 1A), exposed males received an average daily dose of 127 mg/kg/day NAC (Figures 1B,C) and 0.013 mg/kg/day selenium (data not shown). We did not identify any differences in sire body weights between the control and antioxidant treatment groups (Figure 1D).

In rodent models examining type 2 diabetes or diet-induced metabolic syndrome, NAC often improves glucose tolerance and reduces fasting blood glucose levels (Falach-Malik et al., 2016). However, we did not identify any impacts of the paternal antioxidant treatment on sire fasting blood glucose levels or during glucose tolerance testing (Figure 1E). Similarly, Dual-Energy X-ray Absorptiometry (DEXA) scanning did not identify any impacts of the antioxidant treatment on body fat percentage (data not shown).

We next assessed sire glutathione concentrations. As anticipated (Ntamo et al., 2021), NAC treatment increased cellular glutathione levels, including a 50% increase in the testis ( p = 0.0179, Figure 1F). Hepatic mitochondrial DNA (mtDNA) copy number serves as a crude proxy of systemic mitochondrial stress (Castellani et al., 2020). In previous studies, NAC treatment ameliorated alcohol-induced increases in hepatic mitochondrial stress, including increases in mtDNA (Caro et al., 2014). Unexpectedly, we identified modest evidence of increased mitochondrial stress in antioxidant-treated males, which exhibited an 80% increase in mtDNA copy number compared to controls (Figure 1G).

### 3.2 Preconception male antioxidant supplementation modifies female placental histological patterning

Of the eight exposed males in each treatment, four control males and five antioxidant-treated males sired litters, which we used in our comparisons. After diagnosing pregnancy on gestational day ten, we ceased all animal handling and left dams undisturbed until gestational day 16.5 (GD16.5). We then sacrificed pregnant dams, excised the female reproductive tract, and collected multiple measures of offspring fetoplacental growth. As in our previous studies, we selected GD16.5, as this time point represents the phase of pregnancy during which placental growth (in terms of diameter, thickness, and weight) has plateaued, while fetal growth continues to increase (Coan et al., 2004; Mu et al., 2008).

**FIGURE 2:**
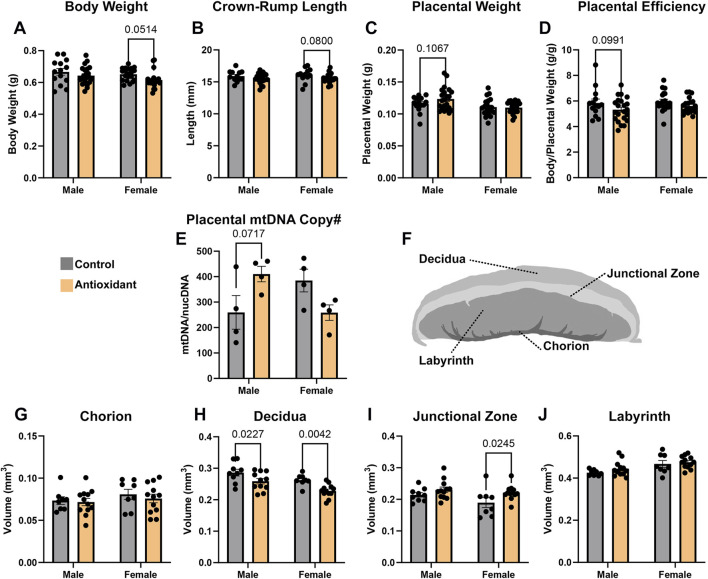
Chronic high-dose antioxidant supplementation modifies offspring placental histological patterning. We used a linear mixed model with repeated measures, followed by a two-way ANOVA, to compare the combined effects of preconception paternal antioxidant treatment on male and female offspring **(A)** fetal weights, **(B)** crown-rump lengths, **(C)** placental weights, and **(D)** placental efficiency (n = 4 to 5 litters). **(E)** We used qPCR to compare placental mitochondrial DNA copy number in male and female placentae derived from each treatment group (n = 4). **(F)** Schematic diagram depicting the histological layers of the murine placenta. Using phosphotungstic acid staining to enhance tissue contrast and micro-CT imaging, we compared the proportional volumes of the placental **(G)** chorion, **(H)** decidua, **(I)** junctional zone, and **(J)** labyrinth in male and female offspring sired by males from the control and antioxidant preconception treatment groups. We used a two-way ANOVA followed by either Sidak’s or Tukey’s *post hoc* testing to compare treatment and sex; data represent mean ± SEM.

We first used a linear mixed model to compare measures of fetoplacental growth between treatments and then followed these analyses with a two-way ANOVA to contrast the effects of the preconception antioxidant treatments and offspring sex. We observed a modest 5% decline in female offspring body weight ( p = 0.0514), but no differences in male offspring (Figure 2A). We did not observe any effects of paternal antioxidant supplementation on crown-rump lengths, placental weights, or placental efficiency in either male or female offspring (Figures 2B–D). We did not observe any treatment effects on gestation length, litter size, or offspring sex ratio among the experimental treatments (data not shown).

As a rough measure of mitochondrial health, we used qPCR to assay mtDNA copy number in the placenta. We did not observe any differences in mtDNA copy number in either male or female placentae (Figure 2E). We next used micro-CT imaging to determine the impacts of chronic paternal antioxidant exposure on placental patterning and histological organization. This technique enables the three-dimensional quantification of the murine placenta, allowing for discrimination and proportional comparisons of the placental chorion, labyrinth, junctional zone, and decidua layers (Figure 2F) (Lesciotto et al., 2020; Clercq et al., 2019). This analysis revealed ∼10%–15% decreases in the proportional volume of male and female decidua, respectively ( p = 0.0227 and p = 0.0042), and a 15% increase in the proportional volume of the female junctional zone ( p = 0.0245). We did not identify any differences in the proportional volume of the placental chorion or labyrinth layers (Figures 2G–J).

### 3.3 Preconceptional male antioxidant supplementation modifies offspring craniofacial shape and symmetry

To determine the effects of chronic paternal antioxidant supplementation on craniofacial growth and patterning, we employed geometric morphometrics. We then performed a Procrustes ANOVA and identified strong evidence of changes in the shape of the left and front facial profiles, but did not identify any changes in the right profile (Supplementary Table S3). We did not identify evidence of differences in centroid size for any of the profiles.

**FIGURE 3:**
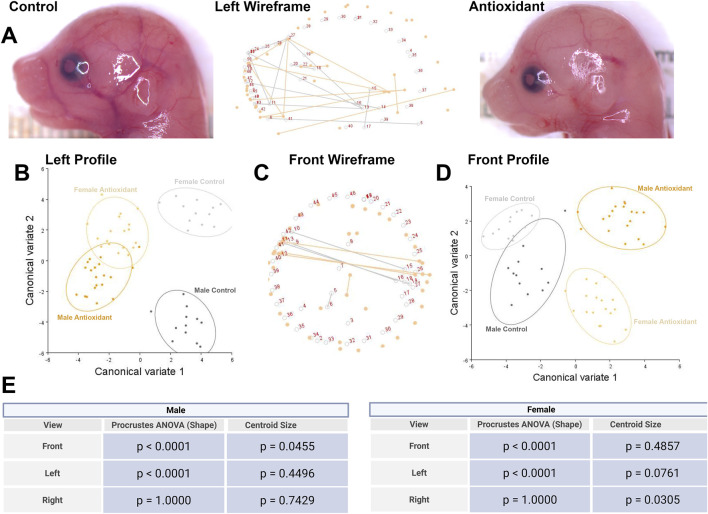
Preconception paternal antioxidant supplementation alters offspring craniofacial shape and symmetry. We employed geometric morphometrics and Procrustes analysis of variance (ANOVA), followed by canonical variate (CV) analysis, to evaluate the effects of paternal antioxidant treatment on facial shape and symmetry. **(A)** Representative images of male offspring derived from control (left) and antioxidant-treated males (right) fetuses flanking a wireframe graph of CV1 (center), illustrating the relative shifts in facial landmarks in male offspring. **(B)** CV plot depicting treatment-induced changes in the left facial profile. **(C)** Wireframe graph of CV1 and **(D)** CV plot depicting treatment-induced changes in the front facial profile. We used Procrustes ANOVA to evaluate the effects of paternal antioxidant supplementation on the facial shape and symmetry of **(E)** male and female offspring.

We then conducted canonical variate (CV) analysis, which identified alterations in the growth of the jaw and positioning of the eyes and ears (Figure 3A). CV analysis of geometric facial relationships in the left profile revealed paternal antioxidant supplementation induced a morphometric shift away from the control treatment along canonical variate one and to a lesser extent, canonical variate two, which together accounted for approximately 45% and 41% of the observed variance in our model (Figure 3B). Similarly, analysis of the front profile revealed shifts in canonical variates one and two, with a shift of midline features to the left (Figures 3C,D).

We then used the raw CV scores to conduct three independent multivariate analyses, including MANOVA, ANOSIM, and PERMANOVA, followed by Bonferroni correction to identify significant differences in clustering and distance between treatment groups. Each of these statistical tests revealed strong evidence ( p < 0.0006) of pairwise differences between the treatment groups (Supplementary Table S4).

When we separated males and females and performed a Procrustes ANOVA, we identified an impact of paternal antioxidant supplementation on centroid size for the male front profile and female left and right profiles, indicating a shift in facial size independent of shape (Figure 3E). Procrustes ANOVA also identified changes in the shape of the left and front profiles of male and female offspring, but not the right (Figure 3E). Given the observed shape differences on the left but not the right profiles, we next used MorphoJ to analyze symmetrical differences in the shape of the frontal facial profile using a set of symmetrical landmarks between the left and right sides of the face, then conducted Procrustes shape ANOVA and a shape MANOVA. Procrustes ANOVA identified a treatment effect on overall symmetrical shape differences in male and female offspring (p = 0.0013 and p = 0.0052, respectively). In contrast, a shape MANOVA did not find evidence of a treatment effect on the symmetric component of shape variation (male p = 0.2641 and female p = 0.1652) (Supplementary Tables S4, S5). These discrepancies may be due to limitations in analyzing 2D images.

### 3.4 Preconceptional male antioxidant supplementation induces sex-specific changes in eye spacing and head size

**FIGURE 4:**
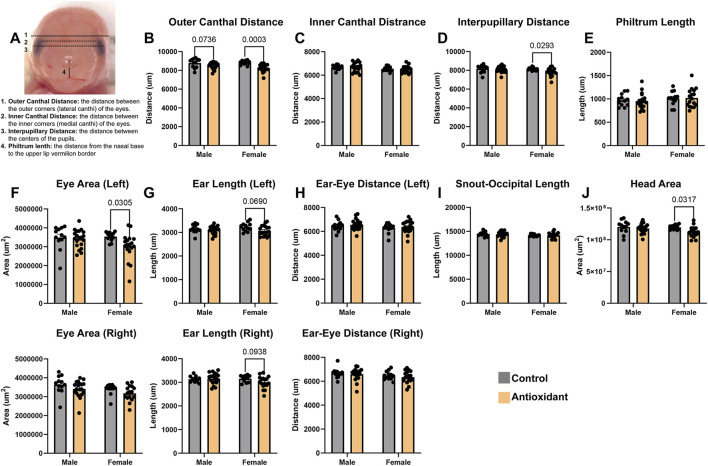
Preconception paternal antioxidant supplementation exerts sex-specific changes in linear measurements of offspring eye spacing and head size. **(A)** Graphic representation of the employed measures of craniofacial morphology in the frontal profile, which are established measures disrupted in mouse models examining prenatal alcohol exposure (Anthony et al., 2010). We used a two-way ANOVA followed by Tukey’s *post hoc* testing to determine the effects of the paternal antioxidant treatment on **(B)** outer canthal distance, **(C)** inner canthal distance, **(D)** interpupillary distance, **(E)** philtrum length **(F)** eye area, **(G)** ear length, **(H)** ear-eye distance, **(I)** snout-occipital length, and **(J)** head area. Data represent mean ± SEM.

We then compared linear measures utilizing established facial measurements routinely employed in studies examining mouse models of craniofacial dysgenesis (Figure 4A) (Anthony et al., 2010). We observed strong evidence for an effect of paternal antioxidant supplementation on female outer canthal distance, with the offspring of antioxidant-exposed fathers displaying a 6.5% reduction ( p = 0.0003, Figure 4B). We also identified a 4% reduction in female interpupillary distance ( p = 0.0293), but did not find evidence to support effects on inner canthal distance or philtrum length (Figures 4C–E).

In the female offspring of antioxidant-treated males, we observed a 15% reduction in the area of the left eye, but did not identify any differences in the right ( p = 0.0305, Figure 4F). We did not identify any differences in the left or right ear length, or ear-eye distance in either male or female offspring (Figures 4G,H). We did not identify any differences in snout-occipital length for either male or female offspring, but did identify a 6% decrease in head area for female offspring ( p = 0.0317, Figures 4I,J).

## 4 Discussion

Recent studies strongly indicate that antioxidant supplementation alone modifies the sperm epigenome, suggesting disruptions in male redox health, generally, may induce paternal effects on offspring development (*reviewed* (Moazamian et al., 2025)). Herein, we sought to determine if a chronic antioxidant regimen could induce paternal effects on offspring fetoplacental growth and craniofacial development. Our findings reveal that chronic paternal antioxidant treatment induced sex-specific effects on offspring placental histological patterning. More strikingly, our work also reveals that administration of antioxidants to ostensibly non-stressed males alters craniofacial shape and symmetry in both male and female offspring, suggesting that chronic redox interventions themselves carry an intergenerational liability.

N-acetylcysteine (NAC) is a widely used antioxidant with therapeutic applications across multiple health conditions. It is also commonly taken as a dietary supplement to support athletic performance, cognitive function, and general wellness (Schwalfenberg, 2021). Although the NAC doses used here are higher than those typically administered in humans, this difference reflects the much faster clearance rate of NAC in mice (half-life ≈34 min in mice vs. 2.27 h in humans) (Borgström et al., 1986; Zhou et al., 2015). Accordingly, our dosing is lower than that in prior studies in mice, which used 250–1,000 mg/kg/day to model therapeutic effects against oxidative stress, teratogenicity, and neurobehavioral deficits (Charron et al., 2020; Parnell et al., 2010; Marian et al., 2006; Wright et al., 2015).

Despite its broad use, a growing body of evidence indicates that NAC may disrupt normal physiology in non-stressed systems. For instance, in otherwise healthy mice, NAC supplementation reduced mitochondrial activity in brown adipose tissue and increased markers of oxidative stress within mitochondria (Peris et al., 2019). In another study, NAC-induced reductive stress, impaired insulin signaling, and glucose transport in muscle and adipose cells of normoglycemic mice, while paradoxically improving both outcomes in diabetic animals (Argaev-Frenkel and Rosenzweig, 2022). These findings suggest that NAC supplementation as a preventive or baseline treatment in non-stressed systems may not be universally beneficial. These preclinical observations align with broader evidence that antioxidant overuse can attenuate physiological adaptations, including those triggered by endurance training, such as increased mitochondrial biogenesis, enhanced cellular defense mechanisms, and improved insulin sensitivity (Merry and Ristow, 2016; Higgins et al., 2020).

While our preliminary study produced several compelling observations, it is important to acknowledge several limitations. First, we cannot definitively determine whether the antioxidant treatment directly affected sperm production and maturation or if the observed paternal effect is a downstream consequence of mitochondrial stress in a distant tissue, such as the liver. Second, although our discussion primarily focused on NAC, we utilized an antioxidant cocktail comprised of both NAC and selenium. Additionally, we included a low dose of the commercial sweetener stevia to enhance palatability, which itself may possess antioxidant properties (Deenadayalan et al., 2021). Therefore, we cannot conclusively determine if the observed antioxidant effects resulted from a single component or a synergistic effect of the combination. Our primary goal, however, was to employ a treatment with the maximal chance of modifying phenotypes that shift in response to chronic paternal alcohol use. Third, our study utilized a C57BL/6J murine model. While highly informative, this strain is known to be sensitive to redox stress and may not fully recapitulate human reproductive biology. Furthermore, our antioxidant treatment was delivered systemically and chronically, a regimen that may differ from the intermittent or more targeted approaches commonly employed in clinical settings. Further, our outcome measures focused predominantly on fetal and placental metrics; therefore, the long-term consequences for postnatal health and aging remain unexplored and warrant further investigation in future studies. Finally, although we suspect that disruptions in redox balance directly underlie the observed paternal effects, it is also plausible that these outcomes arise secondarily from antioxidant-induced metabolic shifts that alter DNA methylation and histone modifications (Chen and Shen, 2025). Moreover, although we presume that the observed paternal effects are transmitted via epigenetic mechanisms, we did not assay any epigenetic measures, including DNA methylation, chromatin organization, or noncoding RNAs. Future studies will compare epigenetic changes in sperm induced by antioxidants to those we observe in alcohol-exposed sperm.

## 5 Conclusion

Although studies in *C. elegans* have shown that mitochondrial stress in the F0 generation can influence bioenergetic function across subsequent generations, evidence for transmissible mitochondrial effects in mammals remains limited (Zhang and Tian, 2022). However, emerging research suggests that similar pathways may operate in mammals, where mitochondrial toxicants or agents that alter the redox environment could have heritable effects on offspring development and health. Our findings extend teratogenic concerns beyond established mitochondrial toxicants like alcohol to include a broader range of environmental exposures and dietary supplements that disrupt redox balance. These results highlight the need for comprehensive preconception counseling for both parents and support expanding epidemiological studies to examine not only impacts on sperm count and fertility but also long-term developmental outcomes in offspring.

## Data Availability

The raw data supporting the conclusions of this article will be made available by the authors, without undue reservation.
