# Supplementary material for: Therapy to teratology: chronic paternal antioxidant supplementation alters offspring placental architecture and craniofacial morphogenesis in a mouse model
Source: Front Cell Dev Biol. 2025 Dec 19;13:1697843. doi: 10.3389/fcell.2025.1697843 (PMC12757331; doi:10.3389/fcell.2025.1697843)
Supplement: Supplementary file 1 [file Table1.docx]

Supplementary Material

| **Number** | **Front View** | **Left/Right View** |
| --- | --- | --- |
| **1** | Highest Head Point | Nose Tip |
| **2** | Bottom of Mandible | Nasion |
| **3** | Right Corner of Mouth | Highest Head Point |
| **4** | Left Corner of Mouth | Curve of Skull |
| **5** | Top of Philtrum | Back of Skull |
| **6** | Bottom of Philtrum | Bottom of Mandible |
| **7** | Nose Tip | Front of Mandible |
| **8** | Nasion | Upper Philtrum |
| **9** | 3 O’clock Eye Position (Left) | Inner Mouth |
| **10** | 12 O’clock Eye Position (Left) | Snout Edge |
| **11** | 9 O’clock Eye Position (Left) | Central Auditory Canal |
| **12** | 6 O’clock Eye Position (Left) | 3 O’clock Eye Position |
| **13** | Pupil Center (Left) | 12 O’clock Eye Position |
| **14** | 3 O’clock Eye Position (Right) | 9 O’clock Eye Position |
| **15** | 12 O’clock Eye Position (Right) | 6 O’clock Eye Position |
| **16** | 9 O’clock Eye Position (Right) | Pupil Center |
| **17** | 6 O’clock Eye Position (Right) |  |
| **18** | Pupil Center (Right) |  |

# Supplemental Table S1. Landmarks used in the morphometric analysis of the front, left, and right facial profiles.

| **Figure** | **Sample Size** | **# Outliers** | **Analysis** |
| --- | --- | --- | --- |
| 1B | CONTROL 8  ANTIOXIDANT 8 | CONTROL 0  ANTIOXIDANT 0 | Two-way ANOVA: Šídák’s multiple comparisons test |
| 1C | CONTROL 8  ANTIOXIDANT 8 | CONTROL 0  ANTIOXIDANT 0 | Unpaired t-test |
| 1D | CONTROL 8  ANTIOXIDANT 8 | CONTROL 0  ANTIOXIDANT 0 | Mixed-effects analysis: Dunnett’s multiple comparisons test |
| 1E | CONTROL 8  ANTIOXIDANT 8 | CONTROL 0  ANTIOXIDANT 0 | AUC, Two-way ANOVA: Dunnett’s multiple comparisons test |
| F | CONTROL 8  ANTIOXIDANT 8 | CONTROL 0  ANTIOXIDANT 0 | Unpaired t-test |
| G | CONTROL 8  ANTIOXIDANT 8 | CONTROL 0  ANTIOXIDANT 0 | Unpaired t-test |
| 2A - D | CONTROL 14 males, 20 females; 4 litters  ANTIOXIDANT 22 males, 21 females; 5 litters | CONTROL 0  ANTIOXIDANT 0 | Linear mixed model, Mixed-effects analysis: Šídák’s multiple comparisons test |
| 2E | CONTROL 4 males, 4 females; 4 litters  ANTIOXIDANT 4 males, 4 females; 4 litters | CONTROL 0  ANTIOXIDANT 0 | Two-way ANOVA: Tukey’s multiple comparisons test |
| 2G - J | CONTROL 9 males, 8 females; 4 litters  ANTIOXIDANT 12 males, 12 females; 5 litters | CONTROL 0  ANTIOXIDANT 0 | Two-way ANOVA: Tukey’s multiple comparisons test |
| 3B, D | CONTROL 12 males, 12 females; 3 litters  ANTIOXIDANT 20 males, 17 females; 4 litters | CONTROL 0  ANTIOXIDANT 0 | Canonical variate analysis |
| 3E | CONTROL 12 males, 12 females; 3 litters  ANTIOXIDANT 20 males, 17 females; 4 litters | CONTROL 0  ANTIOXIDANT 0 | Procrustes ANOVA |
| 4A - J | CONTROL 12 males, 12 females; 3 litters  ANTIOXIDANT 20 males, 17 females; 4 litters | CONTROL 0  ANTIOXIDANT 0 | Two-way ANOVA: Tukey’s multiple comparisons test |

**Supplemental Table S2. Detailed descriptions of the statistical tests and the sample sizes employed in this study.**

**Supplemental Table S3. Results of the Procrustes ANOVA and pairwise analyses of canonical variant scores.**

PROCRUSTES ANOVA RESULTS

| View | Procrustes ANOVA p-value | Centroid p-value |
| --- | --- | --- |
| Male Front | p < 0.0001 | p = 0.0455 |
| Male Left | p < 0.0001 | p = 0.4496 |
| Male Right | p = 1.0000 | p = 0.7429 |
| Female Front | p < 0.0001 | p = 0.4857 |
| Female Left | p < 0.0001 | p = 0.0761 |
| Female Right | p = 1.0000 | p = 0.0305 |

MANOVA RESULTS, FRONT (Bonferroni Corrected)

|  | Control Female | Control Male | Antioxidant Female | Antioxidant Male |
| --- | --- | --- | --- | --- |
| Control Female |  | 2.9513E-08 | 5.8773E-14 | 1.1108E-14 |
| Control Male | 2.9513E-08 |  | 1.0199E-11 | 2.8126E-14 |
| Antioxidant Female | 5.8773E-14 | 1.0199E-11 |  | 3.0212E-15 |
| Antioxidant Male | 1.1108E-14 | 2.8126E-14 | 3.0212E-15 |  |

ONE-WAY ANOSIM, FRONT (Bonferroni Corrected)

|  | Control Female | Control Male | Antioxidant Female | Antioxidant Male |
| --- | --- | --- | --- | --- |
| Control Female |  | 0.0006 | 0.0006 | 0.0006 |
| Control Male | 0.0006 |  | 0.0006 | 0.0006 |
| Antioxidant Female | 0.0006 | 0.0006 |  | 0.0006 |
| Antioxidant Male | 0.0006 | 0.0006 | 0.0006 |  |

ONE-WAY PERMANOVA, FRONT (Bonferroni Corrected)

|  | Control Female | Control Male | Antioxidant Female | Antioxidant Male |
| --- | --- | --- | --- | --- |
| Control Female |  | 0.0006 | 0.0006 | 0.0006 |
| Control Male | 0.0006 |  | 0.0006 | 0.0006 |
| Antioxidant Female | 0.0006 | 0.0006 |  | 0.0006 |
| Antioxidant Male | 0.0006 | 0.0006 | 0.0006 |  |

MANOVA RESULTS, LEFT (Bonferroni Corrected)

|  | Control Female | Control Male | Antioxidant Female | Antioxidant Male |
| --- | --- | --- | --- | --- |
| Control Female |  | 1.1084E-11 | 7.3072E-13 | 6.1209E-16 |
| Control Male | 1.1084E-11 |  | 8.8924E-15 | 9.4514E-15 |
| Antioxidant Female | 7.3072E-13 | 8.8924E-15 |  | 2.7162E-13 |
| Antioxidant Male | 6.1209E-16 | 9.4514E-15 | 2.7162E-13 |  |

ONE-WAY ANOSIM, LEFT (Bonferroni Corrected)

|  | Control Female | Control Male | Antioxidant Female | Antioxidant Male |
| --- | --- | --- | --- | --- |
| Control Female |  | 0.0006 | 0.0006 | 0.0006 |
| Control Male | 0.0006 |  | 0.0006 | 0.0006 |
| Antioxidant Female | 0.0006 | 0.0006 |  | 0.0006 |
| Antioxidant Male | 0.0006 | 0.0006 | 0.0006 |  |

ONE-WAY PERMANOVA, LEFT (Bonferroni Corrected)

|  | Control Female | Control Male | Antioxidant Female | Antioxidant Male |
| --- | --- | --- | --- | --- |
| Control Female |  | 0.0006 | 0.0006 | 0.0006 |
| Control Male | 0.0006 |  | 0.0006 | 0.0006 |
| Antioxidant Female | 0.0006 | 0.0006 |  | 0.0006 |
| Antioxidant Male | 0.0006 | 0.0006 | 0.0006 |  |

MANOVA RESULTS, RIGHT (Bonferroni Corrected)

|  | Control Female | Control Male | Antioxidant Female | Antioxidant Male |
| --- | --- | --- | --- | --- |
| Control Female |  | 3.1295E-11 | 1.7522E-12 | 1.9128E-11 |
| Control Male | 3.1295E-11 |  | 9.3827E-14 | 2.2414E-12 |
| Antioxidant Female | 1.7522E-12 | 9.3827E-14 |  | 5.3528E-13 |
| Antioxidant Male | 1.9128E-11 | 2.2414E-12 | 5.3528E-13 |  |

ONE-WAY ANOSIM, RIGHT (Bonferroni Corrected)

|  | Control Female | Control Male | Antioxidant Female | Antioxidant Male |
| --- | --- | --- | --- | --- |
| Control Female |  | 0.0006 | 0.0006 | 0.0006 |
| Control Male | 0.0006 |  | 0.0006 | 0.0006 |
| Antioxidant Female | 0.0006 | 0.0006 |  | 0.0006 |
| Antioxidant Male | 0.0006 | 0.0006 | 0.0006 |  |

ONE-WAY PERMANOVA, RIGHT(Bonferroni Corrected)

|  | Control Female | Control Male | Antioxidant Female | Antioxidant Male |
| --- | --- | --- | --- | --- |
| Control Female |  | 0.0006 | 0.0006 | 0.0006 |
| Control Male | 0.0006 |  | 0.0006 | 0.0006 |
| Antioxidant Female | 0.0006 | 0.0006 |  | 0.0006 |
| Antioxidant Male | 0.0006 | 0.0006 | 0.0006 |  |

**Supplemental Table S4** Analysis of asymmetry – males

**Supplemental Table S5** Analysis of asymmetry – females
